# Surgical efficacy and survival outcomes of fluorescein sodium-guided surgery in glioblastoma: a single-center experience

**DOI:** 10.1016/j.bas.2025.105597

**Published:** 2025-09-06

**Authors:** Patrick Kuppler, Claudia Ditz, Christina Richter, Maria Vittoria Matone, Jakob Matschke, Anastassia Löser, Peter Schramm, Naureen Keric, Jan Leppert

**Affiliations:** a University Hospital of Schleswig-Holstein, Campus Lübeck, Department of Neurosurgery, Lübeck, Germany; b Bundeswehr Hospital Westerstede, Department of Neurosurgery, Westerstede, Germany; c University Medical Center Hamburg-Eppendorf, Institute of Neuropathology, Hamburg, Germany; d University Hospital of Schleswig-Holstein, Campus Lübeck, Department of Radiooncology, Lübeck, Germany; e University Hospital of Schleswig-Holstein, Campus Lübeck, Department of Neuroradiology, Lübeck, Germany

**Keywords:** Fluorescein sodium (FS), Fluorescence-guided resection, Glioblastoma, Gross total resection, Neuro-oncology, Tumor margin visualization

## Abstract

**Introduction:**

Fluorescence-guided (FG) surgery enhances the extent of resection (EOR) in glioblastoma. While 5-aminolevulinic acid (5-ALA) is the current standard, fluorescein sodium (FS) has emerged as a promising, accessible alternative. This study evaluates the impact of FS-guided resection on surgical outcomes and survival compared to conventional white-light (WL) resection.

**Research question:**

Does FS-guided surgery improve EOR, overall survival (OS), and progression-free survival (PFS) compared to WL-guided glioblastoma surgery?

**Material and methods:**

In this retrospective single-center analysis, data from patients with newly diagnosed glioblastoma who underwent primary resection with gross total resection (GTR) intent between 2013 and 2023 were evaluated. Based on intraoperative technique, patients were allocated to either FS-guided or WL-guided surgery groups. Logistic and linear regression assessed FS impact on GTR and residual tumor volume. Multivariate Cox regression and Kaplan-Meier analysis were used to assess OS and PFS.

**Results:**

Among 128 patients, 85 (66 %) underwent FS-guided surgery. GTR was achieved in 69 % of FS cases versus 36 % with WL (p = 0.0004; OR = 1.379). FS significantly reduced residual tumor volume (1.82 ml vs. 8.83 ml; p = 0.0003). MGMT methylation, GTR, and Stupp-protocol therapy were identified as independent predictors of improved OS and PFS. FS use itself showed no significant association with survival outcomes.

**Discussion and conclusion:**

FS-guided surgery significantly improved EOR and reduced residual tumor volume without impacting OS or PFS. It represents a safe, effective alternative to standard WL resection. Prospective studies should explore combining fluorophores for optimal resection strategies.

## Highlights

• Fluorescein sodium (FS)-guided surgery significantly improves gross total resection (GTR) in glioblastoma.
• Residual tumor volume is reduced nearly fivefold with FS compared to white-light surgery.
• No survival benefit observed for FS-guided resection despite improved resection outcomes.
• GTR, MGMT methylation, and Stupp-protocol remain strong predictors of OS and PFS.
• FS enables real-time tumor visualization and seamless workflow during resection.
• FS is safe, well tolerated, and a cost-effective alternative to 5-ALA in glioma surgery.
• Combining FS with 5-ALA may further enhance resection and survival outcomes.

## 1 Introduction

Surgical resection is a crucial prognostic factor in the treatment of glioblastoma patients as a greater extent of resection (EOR) has not only been associated with better overall survival (OS) and progression-free survival (PFS), but also with an improved quality of life (Aldave et al., 2013; Brown et al., 2016; Lacroix et al., 2001; Sanai et al., 2011). However, due to the infiltrative nature of glioblastomas, intraoperative differentiation of tumor tissue from normal parenchyma remains a significant surgical challenge. Especially at the invasive tumor margins visual or tactile differentiation of the tissue is often not possible.

In this context, fluorescence-guided surgery (FGS) has emerged as a useful tool to enhance surgical precision. The most commonly used fluorescent agent in glioblastoma surgery is 5-aminolevulinic acid (5-ALA), which is currently considered the gold standard for fluorescence-guided resection based on the results of randomized trials (Stummer et al., 2006). As a natural precursor molecule to the hemeprotein of hemoglobin synthesis 5-ALA is selectively converted into protoporphyrin IX (PpIX) by the tumor cells, which then fluoresces under blue light. This selective uptake by the tumor makes 5-ALA particularly effective in identifying malignant tumor remnants at the resection margins. Numerous studies have shown that, compared to conventional white light (WL) resection, the use of 5-ALA significantly improves the extent of tumor resection and increases PFS and OS rates (Stummer et al., 2006; Coburger et al., 2015; Diez Valle et al., 2014; Gandhi et al., 2019). However, 5-ALA has also several drawbacks such as high costs and phototoxic side effects (Senders et al., 2017). Since the use of 5-ALA requires complete darkness in the operating room, it is often only used at the end of the operation and not during the tumor resection in order not to jeopardize the visualization of blood vessels and other important structures. In addition, the practicality is limited as 5-ALA is administered orally 3–4 h before surgery, which may affect flexibility in preoperative planning. A major limitation of 5-ALA is also its specificity for high grade gliomas. Since it is not reliably absorbed in other brain tumors or metastases, its use in general neuro-oncological surgery is limited (Schupper et al., 2021). For these reasons, fluorescein sodium (FS) is increasingly being used as an alternative in some centers, particularly with regard to cost-effectiveness and safety. FS, which is administered intravenously during surgery, is a non-selective fluorescent agent that is not specifically absorbed by tumor cells, but rather accumulates in tumor regions due to the increased permeability of the blood-brain barrier (BBB). Initial studies on the use of fluorescein show promising results in high grade glioma surgery, with better visualization of tumor boundaries, improved extent of resection (EOR) and prolonged survival (Catapano et al., 2017; Chen et al., 2012; Hong et al., 2019; Katsevman et al., 2020). Recent studies even show that the simultaneous administration of 5-ALA and FS can improve both GTR and OS compared to the use of one of these fluorescent dyes alone (Della Puppa et al., 2019; Pesaresi et al., 2024; Shah et al., 2023; Zeppa et al., 2022).

The aim of this single center study was to assess the impact of FS-guided surgery on the EOR, OS, and PFS in patients with newly diagnosed glioblastoma, compared to conventional WL surgery. This study investigates whether FS may serve as a useful adjunct to current surgical approaches, particularly with regard to improving GTR rates and influencing long-term survival outcomes.

## 2 Patients and methods

### 2.1 Study design and data collection

This retrospective study was conducted at a single institution, analyzing data from patients with newly diagnosed glioblastoma who underwent primary tumor resection between 2013 and 2023. The study was approved by the local Ethics Committee (reference 20–260) of the University of Lübeck, Germany. Patient informed consent was not required. Patient inclusion criteria were: 1) newly diagnosed, untreated and histologically confirmed glioblastoma; 2) the presence of a preoperative, and a postoperative MRI study acquired no later than 48 h after the operation; 3) preoperative treatment indication of GTR; 4) tumor location distant from critical functional areas. Patients were excluded in case of: 1) biopsy only; 2) partial resection; 3) unknown extent of resection; 4) missing information on the type of surgical technique (fluorescence-guided or WL resection). Complete resectability of the tumor was assessed independently by two experienced neurosurgeons based on the preoperative MRI scans. The intended surgical objective was classified as either GTR or partial resection/biopsy. The included patients were divided into two groups depending on the surgical technique used: the FS group, in which FS-guided surgery was performed, and the control group, in which conventional WL resection was performed. FS-guided resections were gradually introduced starting in 2016, whereas WL resections were performed continuously from 2013 to 2023. Thus, FS was more frequently used in recent years. Clinical data, including baseline patient characteristics such as age, sex, Karnofsky performance score (KPS), and recursive partitioning analysis (RPA) were collected from the patient records. KPS was dichotomized with a cut-off of 70 % (<70 % vs ≥ 70 %). Information on tumor characteristics, including tumor location and baseline contrast–enhancing tumor volume, as well as type of postoperative oncological treatment, PFS and OS was also recorded. Additionally, molecular markers such as O(6)-methylguanine-DNA methyltransferase (MGMT) methylation status and Ki-67 labeling index were analyzed. Surgical parameters, including the extent of resection (EOR), operative time and postoperative residual tumor volume, were also evaluated.

### 2.2 Quantitative assessment of tumor volume

Quantitative assessment of gadolinium contrast–enhancing tumor volume was performed using the pre- and postoperative MRI scans. The tumor volume was manually delineated and computed using the software “Brainlab” (Brainlab AG, Munich, Germany). Both baseline contrast–enhancing tumor volume and postoperative residual tumor volume were assessed. In case of multifocal disease, all tumor locations were measured and the volumes totaled. Postoperative blood products, which are hyperintense in the acute phase on non-contrast-enhanced T1-weighted sequences were excluded.

### 2.3 Surgical management

**Fig. 1:**
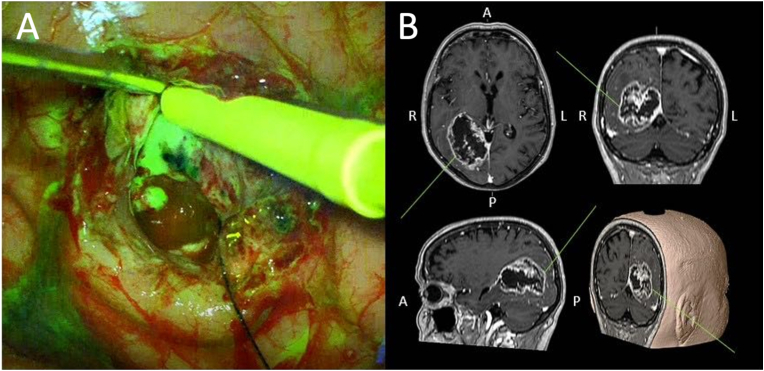
Resection of fluorescent glioblastoma tissue using an ultrasonic aspirator in a subcortical region (A), guided by axial, sagittal, coronal, and 3D-reconstructed MRI navigation (B).

All patients were treated with the aim of achieving GTR of the contrast-enhanced tumor volume and all surgeries were performed with the use of image-guided navigation. In the FS-group, FS (Fluorescein ALCON® 10 %, Novartis AG, Basel, Switzerland) was administered at a low dose of 2 mg/kg after induction of anesthesia immediately before positioning the patient. Surgery was performed with an KINEVO® 900 microscope (Carl Zeiss Meditec AG, Jena, Germany) equipped with a YELLOW 560 filter, which enables the identification of fluorescent tissue (Fig. 1). Fluorescent to WL illumination was used alternately during tumor debulking and resection in the FS-group. After completion of the resection, the edges of the cavity were carefully examined under fluorescent illumination to identify any remaining fluorescent tissue. In the control group, conventional WL resection was performed using standard neurosurgical techniques without fluorescence assistance. All operations were performed by experienced neurosurgeons specialized in brain tumor resection. Intraoperative adjuncts, including neuronavigation and standard neuromonitoring (motor evoked potentials and brain mapping when indicated), were used in both groups. All patients received standardized post-craniotomy care. Magnetic resonance imaging (MRI) was performed in all patients within 48 h of surgery.

### 2.4 Endpoints and outcome measures

The primary endpoint of this analysis was the difference in EOR and postoperative residual contrast-enhancing tumor volume between the two groups as measured by postoperative MRI scans. Regarding the EOR, patients were categorized as having “no residual tumor”, defined as GTR or having “measurable residual tumor”, defined as subtotal resection (STR). The amount of residual contrast-enhancing tumor was assessed volumetrically. Secondary outcomes evaluated in this study included OS, PFS, and duration of surgery. OS was defined as the time from initial surgery to the date of death from any cause in month. PFS was calculated by the time from initial surgery to the first documented disease progression detected on follow-up MRI scans. Follow-up resections or salvage interventions were recorded where applicable. One patient in the WL group underwent an early re-resection due to residual tumor. No other salvage therapies were applied during the initial treatment phase. The relationship of other risk factors to clinical outcome were also analyzed.

### 2.5 Statistical analysis

Data analysis was performed using the software RStudio (Posit Team (2024): Integrated Development Environment for R. Posit Software, PBC, Boston, MA, USA). Descriptive statistics were performed to compare demographic, clinical, radiological, and molecular characteristics between the two groups. Categorical variables, such as sex, tumor side, MGMT methylation status, and the degree of resection (STR vs. GTR), were analyzed using the Chi-square test or Fisher's exact test, as appropriate. Continuous variables, including age, tumor volume, operative time, and postoperative residual tumor volume, were assessed for normality and compared using either the independent two-sample *t*-test (for normally distributed data) or the Mann-Whitney *U* test (for non-normally distributed data). To assess the impact of FS-guided surgery on the likelihood of achieving GTR, linear and logistic regression models were used. These models adjusted for potential confounding variables, including tumor size, location, and molecular markers. For survival analyses, multivariate Cox regression models were applied to evaluate the influence of surgical technique, MGMT methylation status, GTR, and adjuvant treatment on OS and PFS. Univariate survival analyses were performed using Kaplan-Meier survival curves and compared using the log-rank test. An independent two-sample *t*-test was used to compare the duration of surgery between the two groups. Statistical significance was defined as p < 0.05. Box-Whisker-Plots show median (band), first and third quartiles (box) and 1.5-fold IQR (whisker).

## 3 Results

### 3.1 Study population and group comparison

**Fig. 2:**
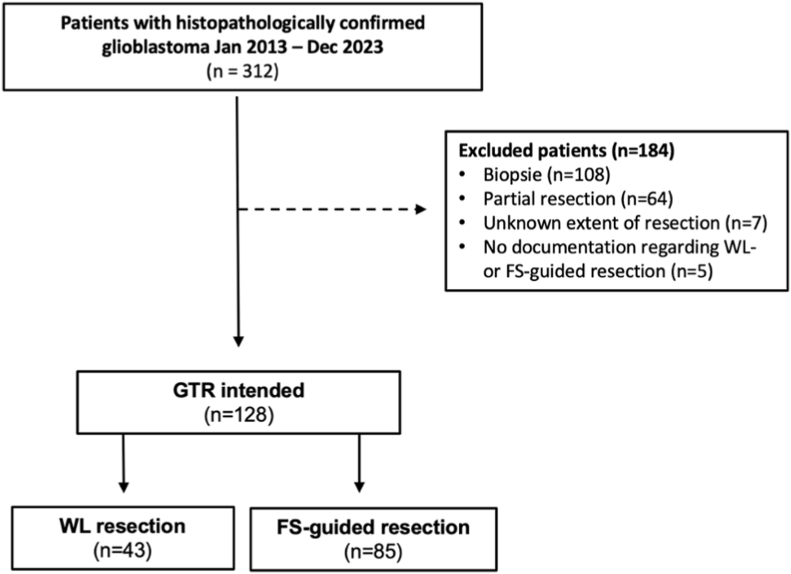
Flowchart illustrating the selection process for the 2 study groups.

A total of 312 patients with newly-diagnosed and histopathologically confirmed glioblastoma were identified at the university hospital Schleswig-Holstein, Campus Lübeck during the study period. 108 patients were excluded who underwent biopsy, 64 patients who underwent partial resection, and 7 patients for whom the planned extent of resection was not documented. In 5 excluded patients, the type of resection (WL or FS-guided resection) was not documented. Finally, 128 glioblastoma patients with intended GTR were analyzed. Of these, 85 patients (66 %) underwent surgery with FS guidance, while 43 patients (34 %) underwent conventional WL resection. The recruitment process is shown in Fig. 2. The characteristics of the study cohort as well as a comparison of characteristics and surgical outcomes between groups is given in Table 1.

**Table 1:** Characteristics and surgical outcomes of the study cohort. Entire cohort and subgroups are showed separately.

Parameter	Entire cohort (n = 128)	WL resection (n = 43 [34 %])	FS-guided resection (n = 85 [66 %])	P value (<0.001)
**Demographic**				
Age (years), mean (IQR)	62 (55–70)	64 (55–75)	62 (56–68)	0.237
Age categories, n (%)				
≤60 years	55 (43)	16 (37)	55 (43)	0.455
>60 years	73 (57)	27 (63)	73 (57)	
Sex, n (%)				
Female	60 (47)	23 (53)	37 (44)	0.379
Male	68 (53)	20 (47)	48 (56)	
**Clinical**				
KPS, n (%)				
≤70 %	16 (13)	5 (12)	11 (13)	1.0
>70 %	112 (87)	38 (88)	74 (87)	
Postoperative neurological deficit, n (%)				
Yes	18 (14)	8(19)	10 (12)	0.434
No	110 (86)	35 (81)	75 (88)	
Postoperative treatment, n (%)				
Stupp-protocol	101 (79)	32 (74)	96 (81)	0.512
Other/None	27 (21)	11 (26)	16 (19)	
**Radiological**				
Tumor side, n (%)				
Left hemisphere	50 (39)	20 (47)	30 (35)	0.386
Right hemisphere	77 (60)	23 (53)	54 (64)	
Both hemispheres	1 (1)	0	1 (1)	
Tumor location, n (%)				
Frontal	33 (25)	17 (40)	16 (18)	0.064
Parietal	14 (11)	5 (11)	9 (10)	
Temporal	57 (44)	12 (28)	45 (52)	
Occipital	14 (11)	5 (11)	9 (10)	
Multi lobular	9 (7)	3 (7)	6 (7)	
Multifocal, n (%)				
Yes	21 (16)	9 (21)	12 (14)	0.47
No	107 (84)	34 (79)	73 (86)	
Initial tumor volume in ml, mean (IQR)	32.9 (12.5–49.8)	35.1 (16.3–49.8)	31.8 (10–49.8)	0.443
**Molecular**				
MGMT methylation, n (%)				
Yes	62 (48)	19 (44)	38 (45)	0.45
No	62 (48)	24 (56)	43 (50)	
Missing	4 (4)	0	4 (5)	
Ki67 labelling index (%), median (IQR)	26.98 (20–30)	27.91 (20–40)	26.56 (20–30)	0.6575
**Surgical outcomes**				
Duration of Surgery in minutes, mean (IQR)	211 (160–250)	223 (186–260)	206 (156–240)	0.117
Degree of resection, n (%)				
STR	53 (41)	27 (64)	26 (31)	<0.001***
GTR	75 (59)	16 (36)	59 (69)	
Postoperative residual tumor volume in ml, mean (IQR)	4.17 (0–3.11)	8.8 (0–103)	1.8 (0–0.6)	0.005**

*WL* white light; *FS* Fluorescein sodium; *IQR* interquartile range; *KPS* Karnofsky performance score; *MGMT* O(6)-methylguanine-DNA methyltransferase; *STR* subtotal resection; *GTR* gross total resection.

In the entire cohort, 68 patients were male (53 %). The mean age was 62 (IQR 55–70) years, with 73 patients (57 %) older than 60 years. The Chi-square test confirmed a significant difference in group sizes (p = 1.52 × 10^−7^), with a higher number of patients in the FS group. Univariate analyses of potential confounding covariables showed no significant group differences in demographic, clinical, radiological, and molecular characteristics, including age (median: 62 vs. 64 years, p = 0.237), sex distribution (p = 0.379), preoperative KPS (p = 1.0), and MGMT methylation status (p = 0.45). No significant differences in preoperative tumor characteristics such as tumor location (p = 0.064) and initial tumor volume (p = 0.443) were observed. While the duration of surgery was similar for WL and FS-guided surgery, the EOR differed significantly between the two groups. FS-guided resection resulted in a higher rate of GTR (69 % vs. 36 %, p < 0.001) and a significantly lower residual tumor volume (1.8 ml vs. 8.8 ml, p = 0.005) compared to WL resection.

### 3.2 Clinical outcomes

The analysis of potential disadvantages of FS-guided surgery revealed no statistically significant difference in the occurrence of postoperative neurological deficits between the FS and WL groups (p = 0.434). Although there was a higher percentage of deficits in the WL group (19 %) than in the FS group (12 %), this difference was not statistically significant. No adverse reactions related to the use of FS were observed in any of the cases.

### 3.3 Extent of resection

**Fig. 3:**
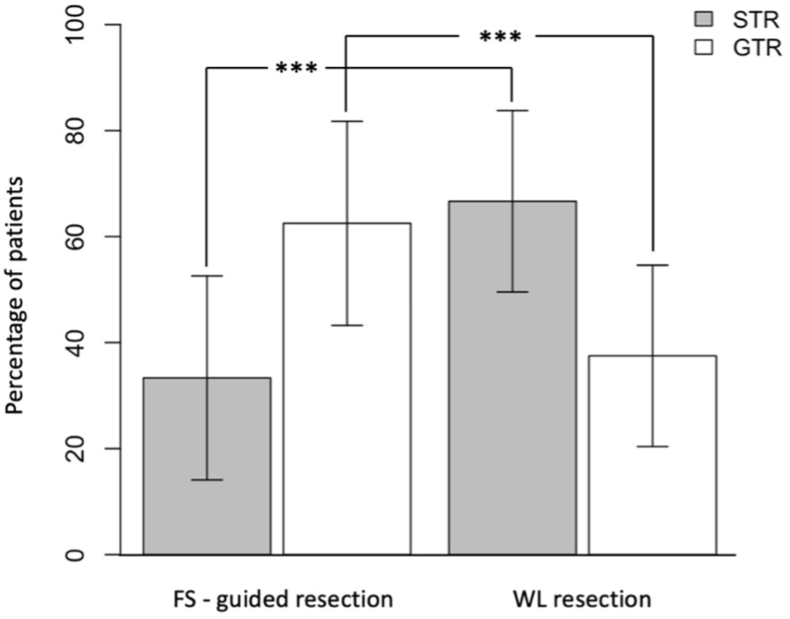
Comparison of the extent of resection (EOR) between fluorescein sodium (FS)-guided surgery and white light (WL) conventional resection. The bar chart illustrates the percentage of patients achieving gross total resection (GTR) or subtotal resection (STR) in the FS and WL group. *** indicates a highly significant difference (logistic regression model: p-value = 0.0004 for GTR; 0.0001 for STR) between the groups. Error bars indicate the standard deviation of the percentages.

The proportion of patients in whom a GTR was achieved was significantly higher in the FS group than in the WL group, as GTR was accomplished in 59 (69 %) of 85 cases in the FS-group and in 16 (36 %) of 43 cases in the WL group (p = 0.0004). This corresponds to a 37.9 % higher probability of achieving a GTR in patients who received FS-guided surgery (odds ratio [OR] = 1.379). A comparison of the EOR between groups is shown in Fig. 3.

### 3.4 Postoperative residual tumor volume and duration of surgery

**Fig. 4:**
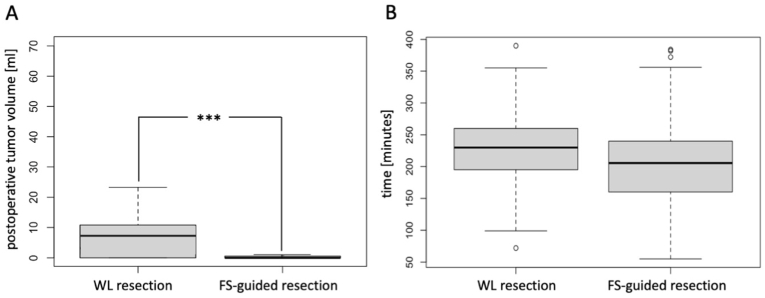
Postoperative residual tumor volume in ml (A) and mean time of surgery in minutes (B) for WL and FS-guided resection. Independent two-sample *t*-test: p = 0.005**, linear regression model: p = 0.0003*** (A); independent two-sample *t*-test: p = 0.1161 (B).

**FS-guided surgery resulted in a significantly lower residual tumor volume compared to conventional WL resection.** The mean residual tumor volume in the FS group was 1.82 ml, compared to 8.83 ml in the WL group (p = 0.005). This corresponds to a 4.8-fold reduction in relative tumor volume after FS-guided surgery (Fig. 4A). The **mean reduction of tumor volume per patient** was **91.17 % (IQR 89.68 % – 100.0 %)** in the WL group and **97.52 % (IQR 99.2 %–100.0 %)** in the FS group (p = 0.0003). With regard to the mean duration of surgery, the operation time in the FS group (206 min (156–240)) was shorter compared to the WL group (223 min (186–260)), but this difference was not statistically significant (p = 0.117; Fig. 4B).

### 3.5 Survival outcomes

**Fig. 5:**
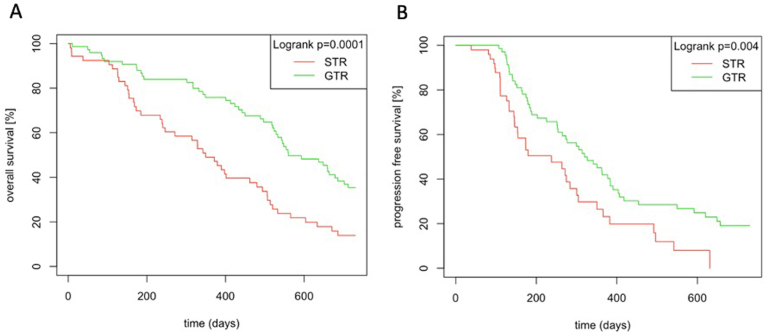
Kaplan-Meier curves illustrating the impact of gross total resection (GTR) versus subtotal resection (STR) on overall survival (OS) (A) and progression-free survival (PFS) (B). Log-rank test results show a significant difference in OS (p = 0.0001) and PFS (p = 0.004) between the GTR and STR group.

**FS-guided surgery** was not found to be an independent predictor of OS or PFS in multivariate analyses. After adjusting for potential confounders, no statistically significant association was observed between FS-guided surgery and OS (*p* = 0.6) or PFS (*p* = 0.2). Consistently, Kaplan–Meier survival curves did not demonstrate a survival benefit for FS-guided surgery compared to white-light (WL) resection. In contrast, several other factors emerged as independent predictors of survival. In terms of surgical outcomes, **GTR** was confirmed as a strong independent predictor of survival, associated with a significantly reduced risk of death (HR 0.20, 95 % CI: 0.095–0.423; *p* < 0.001) and disease progression (HR 0.37, 95 % CI: 0.165–0.820; *p* = 0.015) (Fig. 5). **MGMT promoter methylation** was significantly associated with improved outcomes, with methylated patients showing better OS (hazard ratio [HR] 0.45, 95 % confidence interval [CI]: 0.236–0.861; *p* = 0.018) and PFS (HR 0.42, 95 % CI: 0.214–0.838; *p* = 0.016). **Concomitant radio chemotherapy** according to the **Stupp-protocol** was also linked to favorable survival, significantly improving both OS (HR 0.30, 95 % CI: 0.106–0.832; *p* = 0.023) and PFS (HR 0.24, 95 % CI: 0.066–0.837; *p* = 0.026). Among clinical factors, **sex** was a significant predictor of OS, with **male patients exhibiting a higher mortality risk** compared to females (HR 2.52, 95 % CI: 1.075–5.907; *p* = 0.033). **Age ≥ 60 years** was also associated with significantly worse OS (HR 0.31, 95 % CI: 0.112–0.838; *p* = 0.021), indicating an increased risk of death among older patients. **Postoperative KPS** was significantly associated with PFS in univariate analysis (*p* < 0.0001), though it did not retain significance in the multivariate model (*p* = 0.378). It showed a trend toward influencing OS (*p* = 0.082), suggesting a potential prognostic value. Among radiotherapy-related parameters, receiving a **radiation dose >50 Gy** was strongly associated with improved PFS (HR 0.08; *p* < 0.0001), and a **shorter interval between surgery and radiotherapy** was also linked to longer PFS (HR 0.13; *p* = 0.021). Lastly, **multifocal tumor location** was independently associated with a higher risk of progression (HR 2.7; *p* = 0.029). Other factors, including **Ki67 labeling index, RPA class,** and **time to surgery**, did not show significant associations with OS or PFS in the multivariate analysis. A detailed summary of all evaluated variables is provided in Table 2.

**Table 2:** Univariate (Log-Rank) and multivariate (Cox proportional hazards model) analyses of overall survival (OS) and progression-free survival (PFS) for selected confounding variables. Hazard ratios (HR) with 95 % confidence intervals and corresponding p-values are reported, indicating the impact of each variable on OS and PFS.

	Progression free survival				Overall survival			
	Univariate analysis (Log-Rank)	Multivariate cox proportional hazard regression model			Univariate analysis (Log-Rank)	Multivariate cox proportional hazard regression model		
	p-value	Hazard Ratio [HR]	95 % - Conf. Interv.	p-value	p-value	Hazard Ratio (HR)	95 % - Conf. Interv.	p-value
Age ≥60 years	0.1	1.63	0.656–4.035	0.293	0.2	0.31	0.112–0.838	0.021*
Sex	0.9	2.22	0.972–5.057	0.058	0.8	2.52	1.075–5.907	0.033*
KPS	<0.0001***	0.52	0.117–2.252	0.378	0.2	1.2	0.031–1.231	0.082
RPA	<0.0001***	1.16	0.560–2.401	0.689	0.3	1.01	0.501–2.051	0.969
MGMT methylation	0.05*	0.44	0.183–1.073	0.072	0.0001***	0.25	0.098–0.614	0.003**
Ki67 labeling Index	<0.0001***	1	0.970–1.028	0.926	0.7	1.02	0.987–1.04	0.275
Multifocal location	0.03*	2.7	1.105–6.574	0.029*	0.06	1.87	0.722–4.815	0.198
GTR	<0.0001***	0.13	0.049–0.334	<0.0001***	0.004**	0.22	0.088–0.535	<0.001***
Stupp-protocol	<0.0001***	0.25	0.063–0.944	0.041*	0.005**	0.23	0.048–1.050	0.058
Radiation dose >50Gy	<0.0001***	0.08	0.020–0.287	<0.0001***	0.8	1.76	0.332–9.298	0.507
FS-guidance	0.2	2.56	0.981–6.689	0.055	0.6	1.47	0.534–4.049	0.455
Time to surgery	0.4	0.44	0.165–1.180	0.103	0.2	0.52	0.173–1.532	0.233
Time to RT	0.4	0.13	0.024–0.738	0.021*	0.3	1.44	0.383–5.313	0.588

## 4 Discussion

This study demonstrates that FS-guided surgery significantly improves GTR and reduces the proportion of residual tumor tissue compared to conventional WL resection. These results are consistent with previous retrospective and non-randomized prospective studies investigating the use of FS in high-grade glioma resection. These studies found a higher GTR rate using FS of 73.4–85.7 % vs 30.1–62.5 % compared to WL surgery (Catapano et al., 2017; Chen et al., 2012; Hong et al., 2019; Katsevman et al., 2020; Koc et al., 2008; Shinoda et al., 2003). It is well documented in the literature that the EOR correlates positively with improved survival outcomes (Brown et al., 2016; Lacroix et al., 2001; Karschnia et al., 2023). This study confirmed GTR, defined as no contrast-enhancing residual tumor, as a strong predictor of both PFS and OS in the presented cohort. However, despite the improved resection outcomes associated with the use of FS, no significant impact on OS or PFS was observed in the here presented cohort. This suggests that while FGS increases the EOR and facilitates more complete tumor removal, it does not directly translate into a long-term survival benefit for glioblastoma patients. Interestingly, various previous studies on FS-guided resection have also shown a significant advantage for EOR with this technique, but again, the surgical technique had no significant impact on survival (Katsevman et al., 2020; Koc et al., 2008; Shinoda et al., 2003). Several factors could contribute to this observation. One factor that may influence the observed lack of survival benefit is the complex and heterogeneous nature of glioblastoma, in which tumor recurrence is influenced by a variety of factors, including molecular characteristics, tumor microenvironment and resistance to therapies. With this multifactorial nature of glioblastoma progression, the statistical power may have been too low in smaller studies, including this one. However, there are also indications in the literature that differences between 5-ALA and FS in tumor visualization and margin delineation may contribute to variations in resection quality, which can in turn affect survival outcomes.

Several studies and recent meta-analysis demonstrated not only increased GTR, but also an improvement of OS and PFS with 5-ALA-guided resection compared to conventional WL surgery (Stummer et al., 2006; Diez Valle et al., 2014; Gandhi et al., 2019; Eatz et al., 2022). Similar high-quality studies do not exist for FS and a positive impact of FS-guided resection on survival has not yet been confirmed (Chen et al., 2012). Due to the different distribution pattern of the two fluorophores, the use of 5-ALA could lead to an even more radical tumor resection compared to FS. Likewise, to gadolinium, FS accumulates in tumor margins due to passive diffusion through the sites of BBB breakdown (Musca et al., 2024). Thus, the distribution of FS roughly corresponds to the contrast-enhanced area in the MRI, while the metabolic tracer 5-ALA accumulates in the tumor cells and highlights the infiltrative tumor spread beyond the contrast enhancement.

The visualization of tumor tissue by FS could therefore be limited, especially at the invasive tumor margins. It has been demonstrated that a considerable amount of tumor cells infiltrate around the enhancing region where the BBB has not yet been destroyed (Chang et al., 2007). Recent studies showed that supramaximal resection beyond the boundaries of contrast enhancement provides further survival advantage in high-grade glioma surgery (Glenn et al., 2018). Several studies suggest that the use of 5-ALA is superior for detecting border invasion and tumoral spots within the surgical cavity which facilitates supramaximal resection (Ahrens et al., 2022; Certo et al., 2021; Roberts et al., 2011; Stummer et al., 2014; Suero Molina et al., 2019; Yano et al., 2017). A more radical resection at the infiltrative tumor margins by the use of 5-ALA could therefore lead to a more distinct survival benefit compared to FS-guided surgery. Unfortunately, comparative studies of 5-ALA and FS for high-grade glioma surgery are still limited. In a recent retrospective cohort study on 194 newly diagnosed with high-grade glioma patients treated with either 5-ALA or FS, *Hansen* et al. found no difference in GTR between 5-ALA and FS (64 % vs. 62 % (OR, 0.90, p = 0.76). The median OS was 14.8 months for the 5-ALA group and 19.7 months for the FS group (p = 0.06) (Hansen et al., 2020). The authors conclude that FS can be used as a viable alternative to 5-ALA for intraoperative fluorescent guidance in high-grade glioma surgery. Although 5-ALA is still the only approved drug for fluorescence-guided brain tumor surgery and the available evidence for FS is lower compared to 5-ALA, FS is currently used as an alternative fluorophore in many centers as it offers practical utility advantages, higher cost effectiveness and safe application. However, it should be noted that FS is not officially approved for brain tumor surgery in most countries and is typically used off-label for this purpose. In this study, none of the patients experienced any systemic or local adverse effects associated with the use of FS. Furthermore, FS-guided surgery was not associated with an increased incidence of newly diagnosed postoperative neurological deficits. This finding is in line with existing literature, which describes adverse reactions associated with FS as extremely rare (Restelli et al., 2022). In contrast, adverse effects related to 5-ALA, such as transient elevations of liver enzymes and photosensitivity, have been more frequently reported in clinical studies.

In addition to the above mentioned logistical and economic advantages, the use of FS also offers practical surgical benefits. Intraoperative visualization of 5-ALA under violet–blue filters generally requires a dark operating field, where anatomy and surgical instruments are barely visible. Against this background, tumor resection is largely performed under WL, while blue filters are used to examine the margins of the resection cavity and identify fluorescent tumor remnants. In addition, 5-ALA fluorescence may be hindered by blood, cerebrospinal fluid, cottoned and inadequate illumination of deep working corridors (Della et al., 2023). The use of FS enables a completely different surgical workflow. By alternating the YELLOW 560 filter with WL, FS guidance can be easily utilized during the entire resection time. The improved illumination of the surgical field facilitates the identification of anatomical structures and reduces the risk of accidental injury to vascular or nervous structures. This enables debulking of the tumor and continuous resection of the fluorescent tumor margins under the FS filter using microsurgical or CUSA technique. As resection can be performed during fluorescence visualization without interruptions as required with 5-ALA, FS-guided resection might result in shorter surgery times. In this study, the mean duration of surgery was shorter in the FS group compared to the WL group, but this difference was not statistically significant. Although 5-ALA is considered a highly tumor-specific fluorophore, false positive fluorescence can be observed in normal anatomical structures such as the periventricular area or in the presence of peritumoral inflammatory cells or perifocal edema (La Rocca et al., 2020). In contrast, FS provides a very distinct boundary between fluorescence and non-contrast enhancing brain parenchyma. This, on the contrary, may result in inclusion of areas with blood-brain barrier disruption but without true tumor infiltration, due to its less tumor-specific nature, thus affecting the oncological precision of the resection. Based on observation during the study period, fluorescence caused by mechanical trauma, such as at the corticotomy site, could generally still be distinguished from tumor-associated signal, as it typically appears more diffuse, irregular, and superficial, often emerging with a slight delay after surgical manipulation. In contrast, tumor fluorescence tends to present with a more homogeneous and stable appearance. With sufficient surgical experience and intraoperative correlation, this differentiation can be made with high reliability. Nonetheless, this characteristic of FS still requires a certain level of intraoperative interpretation, and the ability to reliably distinguish between fluorescence patterns may depend on the surgeon's familiarity with the technique.

Another observation during the study period was that low-dose FS (1–4 mg/kg) appears sufficient for clear and distinct visualization of fluorescent tumor margins. Recent studies even suggest an ultra-low-dose of FS (0.5–1 mg/kg) for glioma surgery (Ling et al., 2024). As outlined above, the use of 5-ALA could lead to more radical supramaximal resections, potentially resulting in complications if the tumor is located in near-eloquent areas. Recent studies suggest that FS may be more suitable than 5-ALA for achieving maximum safe resection of near eloquent or eloquent lesions or when the tumor is located in a very deep area (Pesaresi et al., 2024; Xiao et al., 2024). Since 5-ALA and FS differ greatly not only in terms of the properties and mechanisms of action of the agents, but also with regard to the surgical procedure and resection technique, recent studies have investigated the simultaneous use of both fluorophores in high-grade glioma surgery. These initial studies suggest that simultaneous use of 5-ALA and FS may improve both GTR and OS compared to administration of one dye alone (Della Puppa et al., 2019; Shah et al., 2023; Zeppa et al., 2022).

The results of this study support previous evidence that FS is a safe and effective alternative to 5-ALA in the resection of newly diagnosed glioblastomas. In the future, however, FS could also represent a complementary method not only to 5-ALA but also to complementary diagnostic tools, such as confocal laser endomicroscopy (CLE) in order to further optimize the outcomes of FS-guided surgery. CLE, which requires the use of FS, allows for high-resolution, real-time imaging of the tumor margins at a cellular level. Recently, *Abramov* et al. demonstrated that CLE can more accurately identify tumor cells at the margins, even if no residual tumor tissue can be detected with 5-ALA fluorescence (Abramov et al., 2025). The combination of FS for tumor visualization and CLE for detailed tissue analysis could thus represent a powerful synergy in glioblastoma surgery, allowing for better delineation of tumor boundaries and possibly leading to improved survival outcomes, although further studies are needed to confirm this hypothesis.

Limitations of this study include the retrospective design and the potential for selection bias, as patients were not randomized to one or the other surgical technique. As this is a single center analysis with a moderate sample size of 128 patients, the generalizability of the findings and the statistical power of this study are limited. Although the temporal overlap of WL surgery and FSG minimizes systematic bias, FG has been used more frequently in recent years. Improvements in surgical expertise, imaging modalities, and intraoperative adjunct availability over time may have influenced outcomes independently of fluorescence use. Furthermore, potential confounding factors such as surgeon experience, patient comorbidities and complications during the hospital stay, which could influence the patients' outcomes and thus the study's conclusions, have not been addressed in this analysis. Finally, the effects of FS-guided surgery were only investigated in newly diagnosed glioblastoma patients. FS may be inferior to 5-ALA in distinguishing between recurrent glioblastoma and pseudo-progression or radio-necrosis due to the property of FS distribution corresponding to disruption of the BBB. Prospective randomized controlled trials are urgently needed to validate the potential advantages and disadvantages of 5-ALA and FS in direct comparison for high-grade glioma surgery and to explore the potential synergistic effects of combining both 5-ALA and FS in fluorescence-guided surgery and in the further development of intraoperative microscopic imaging technology.

## 5 Conclusions

FL-guided resection is a safe and feasible tool in the surgical treatment of newly diagnosed glioblastomas which, compared to WL resection, significantly improves GTR. A beneficial effect of the increased EOR on patient’ survival is still pending. Prospective validation is required to compare the potential advantages and disadvantages of 5-ALA and SF and to evaluate a potential benefit of simultaneous FG-guided surgery using both fluorophores.

## Declaration of competing interest

The authors declare that they have no known competing financial interests or personal relationships that could have appeared to influence the work reported in this paper.
